# Reproducible Neural Network Simulations: Statistical Methods for Model Validation on the Level of Network Activity Data

**DOI:** 10.3389/fninf.2018.00090

**Published:** 2018-12-19

**Authors:** Robin Gutzen, Michael von Papen, Guido Trensch, Pietro Quaglio, Sonja Grün, Michael Denker

**Affiliations:** ^1^Institute of Neuroscience and Medicine (INM-6) and Institute for Advanced Simulation (IAS-6) and JARA-Institut Brain Structure-Function Relationships (INM-10), Jülich Research Centre, Jülich, Germany; ^2^Theoretical Systems Neurobiology, RWTH Aachen University, Aachen, Germany; ^3^Simulation Lab Neuroscience, Jülich Supercomputing Centre, Institute for Advanced Simulation, JARA, Jülich Research Centre, Jülich, Germany

**Keywords:** spiking neural network, SpiNNaker, validation, reproducibility, statistical analysis, simulation

## Abstract

Computational neuroscience relies on simulations of neural network models to bridge the gap between the theory of neural networks and the experimentally observed activity dynamics in the brain. The rigorous validation of simulation results against reference data is thus an indispensable part of any simulation workflow. Moreover, the availability of different simulation environments and levels of model description require also validation of model implementations against each other to evaluate their equivalence. Despite rapid advances in the formalized description of models, data, and analysis workflows, there is no accepted consensus regarding the terminology and practical implementation of validation workflows in the context of neural simulations. This situation prevents the generic, unbiased comparison between published models, which is a key element of enhancing reproducibility of computational research in neuroscience. In this study, we argue for the establishment of standardized statistical test metrics that enable the quantitative validation of network models on the level of the population dynamics. Despite the importance of validating the elementary components of a simulation, such as single cell dynamics, building networks from validated building blocks does not entail the validity of the simulation on the network scale. Therefore, we introduce a corresponding set of validation tests and present an example workflow that practically demonstrates the iterative model validation of a spiking neural network model against its reproduction on the SpiNNaker neuromorphic hardware system. We formally implement the workflow using a generic Python library that we introduce for validation tests on neural network activity data. Together with the companion study (Trensch et al., 2018), the work presents a consistent definition, formalization, and implementation of the verification and validation process for neural network simulations.

## 1. Introduction

Computational neuroscience is driven by the development of models describing neuronal activity on different temporal and spatial scales, ranging from single cells (e.g., Koch and Segev, 2000; Izhikevich, 2004) to spiking activity in mesoscopic neural networks (e.g., Potjans and Diesmann, 2014; Markram et al., 2015), to whole-brain activity (e.g., Sanz Leon et al., 2013; Schmidt et al., 2018). In order to quantify the accuracy and credibility of the models they must be routinely validated against experimental data. In light of the scarcity of available experimental data, both on the level of structure as well as on the level of activity, making these data available to the community is a high priority for today's neuroscience. This task is being addressed, in particular, by coordinated, large-scale efforts such as the Allen Brain Institute^1^ and the Human Brain Project^2^. However, it is of equal importance that models are delivered in a comprehensible and reproducible form, and that validation is based on standardized statistical tests.

Although there is no general consensus on how models should be described and delivered (Nordlie et al., 2009), a number of frameworks support researchers in documenting and implementing models beyond the level of custom-written code in standard high-level programming languages. These frameworks include guidelines for reproducible network model representations (Nordlie et al., 2009; McDougal et al., 2016), domain-specific model description languages (e.g., Gleeson et al., 2010; Plotnikov et al., 2016), modeling tool-kits (e.g., BMTK^3^, NetPyNE^4^), and generic network simulation frameworks (Davison et al., 2008). To share these models, but also data, with the community several databases and repositories have emerged and are commonly used for this purpose, for example GitHub^5^, OpenSourceBrain^6^, the Neocortical Microcircuit Collaboration Portal^7^ (Ramaswamy et al., 2015), the G-Node Infrastructure (GIN)^8^, ModelDB^9^, NeuroElectro^10^ (Tripathy et al., 2014), or CRCNS ^11^ (Teeters et al., 2008).

The statistical validation of models, however, lacks a standardized approach and supporting software tools. Thus, it is usually open to the authors to define to which degree the simulation outcome is supposed to match the experimental data. In consequence of this *ad hoc* approach, we identify three difficulties encountered with published models:

1. Models are only tested qualitatively instead of quantitatively. For example, the spike trains resulting from the simulation are visually classified (e.g., Voges and Perrinet, 2012), but without calculating specific statistics to quantify the features of the activity. This lack of concrete numbers and detailed records of how the numbers are calculated prevents a direct comparison to other models.
2. The information provided in a publication on the details of how the specific statistical analysis is performed and thus how a model is validated is not sufficient to reproduce the validation scenario.
3. Models are only compared to a single experimental data set using a specific statistical measure. Moreover, the choices of data sets and measures are biased to address specifically the scientific aim of the publication. However, the absence of a standardized procedure to base the validation on a broad set of data sets and statistical measures limits the degree to which confidence in the model is quantified in a context detached from the research conducted in the publication at hand. Moreover, it prevents the direct comparison between published models and their re-use in related studies.

Generic attempts to overcome these difficulties and formalize the validation process include the development of the Python module SciUnit (Omar et al., 2014; Sarma et al., 2016), and the description of workflows for the validation of models (Senk et al., 2017; Kriegeskorte and Douglas, 2018; van Albada et al., 2018). In this study, we build on these efforts in order to introduce a workflow and supporting software to quantitatively compare and validate spiking network models. The provided workflow and software include all necessary analysis steps to ensure reproducibility of the validation process, including the details of extracting the statistical measures.

The validation of spiking neural networks can be performed on two principle levels, which we refer to as “single-cell” and “network” validation. The single-cell scenario assumes that validation of the smallest elements of the circuit leads to realistic emergent dynamics on the network scale (Markram et al., 2015; Reimann et al., 2015). However, the link between the dynamics of the smallest elements and that of larger composite systems is intrinsically complex. Therefore, we argue that the validation process should also include complementary network-level validation, which involves the quantitative comparison of several mono- and bivariate and sometimes higher-order statistics of the spiking activity to capture the complete dynamics of the system.

The advantage of single-cell validation is that the cellular activity, e.g., cellular response to current input, can be well measured in different labs and even under slightly different experimental settings. Network-level validation, on the other hand, is hindered by several aspects. Experimentally, such dynamics can usually only be measured *in-vivo*, which involves more sophisticated experiments than single-cell recordings. Moreover, the large variability between measured systems, e.g., different subjects, can be very large. Such sources of uncertainty need to be taken into account when interpreting the assessed quantitative agreement of the simulation outcome with the experimental reference data.

In this study, we first discuss the concept of validation and introduce the related terminology in Sections 2.1, 2.2. In Sections 2.3 we describe in detail the particular scenario of model-to-model validation, which is the basis of a concrete worked example used for illustration during the remainder of the manuscript. In that example, we quantify the statistical difference between two implementations of the same model, namely the polychronization model (Izhikevich, 2006) and its reproduction on the SpiNNaker neuromorphic hardware system (cf., companion study Trensch et al., 2018). The models, the test statistics, and the formal workflow used for this validation are described in Section 3. In Section 4 we detail how the interplay of different network-level validation tests leads to a quantitative assessment of the SpiNNaker model. Finally, we discuss in Section 5 the conditions under which the models are in acceptable agreement, i.e., for what kind of applications the models are interchangeable. We further discuss the applicability of the proposed workflow for other validation scenarios.

## 2. Validation of Neural Network Simulations

In this section we explore the conceptual background of validation in the context of neural network models by first relating and adapting previously introduced terminology to our domain, and discussing how to draw valid conclusions from this workflow. We then introduce the concept of network-level validation in computational neuroscience; the validation of a simulation on the basis of measures derived from the collective dynamics exhibited by the model. Finally, we discuss the special case of model-to-model validation; the validation of one model implementation against another implementation of the same or a related model.

### 2.1. The Concept of Validation

When considering model simulations and their evaluation, it is important to precisely define the terminology and to be clear about the interpretation of the results in order to judge the validity and the scope of applicability of the model. For all practical purposes, in modeling one should be concerned with its testable correctness relative to the given system of interest, because only this process justifies its use as the basis for analytic reasoning and prediction making. A central aspect in model evaluation is its validation, that is, the process of assigning credibility to a model. Establishing the absolute validity of a model is inherently impossible, as a model is by design an abstraction and simplification of reality (Balci, 1997; Sterman, 2000). Nevertheless, the more aspects of the model are covered by validation tests, the more confidence may be placed into the model in terms of the features exhibited within the limits of an accuracy determined by an acceptable agreement. Thus, there is not a single test that is sufficient for a model to be validated (Forrester and Senge, 1980), and the outcome of a validation process should not be understood as a definite verdict about its validity but as a quantitative evaluation of usefulness and accuracy. This quantification may typically be given in the form of a score, which is either a normalized measure of agreement, or a probability value based on observed evidence and a priori assumptions and beliefs (Carnap, 1968). With the help of such quantified credibility measures, it becomes possible to understand which aspects are well represented by a model, and in consequence, how to weigh and interpret its predictions. Notably, a model thus has a range of applications and a level of description defined by the credibility measures. Stretching the model beyond its intended purpose to a wider range of application would therefore require additional validation tests.

**Figure 1:**
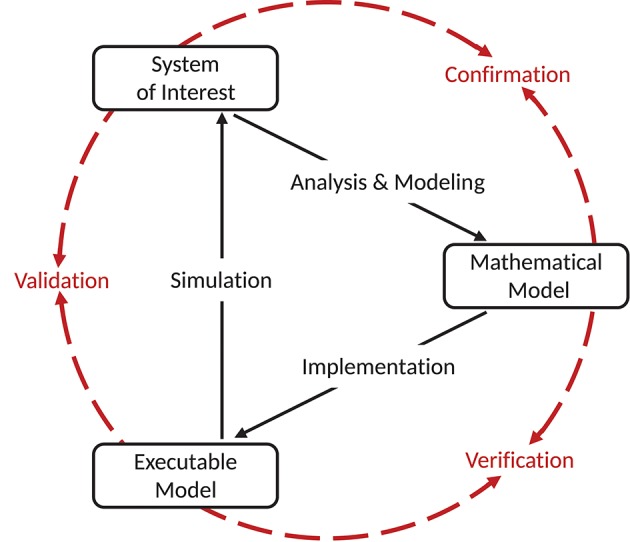
Schematic view of the model simulation environment introduced by Schlesinger (1979). The figure and terminology is adapted from Thacker et al. (2004), and defines the relationships between the system of interest, mathematical model, and executable model as confirmation, verification, and validation. Modeling and simulation activities are indicated by black solid arrows, whereas assessment activities are indicated by red dashed arrows. Figure amended from Trensch et al. (2018).

In 1979 the Technical Committee on Model Credibility of the Society of Computer Simulation established a widely recognized description of a model verification and validation environment. We adapt this terminology to the field of neural network modeling, in line with our companion study (Trensch et al., 2018). The validation setup is separated into three basic elements (see Figure 1). The system of interest can be defined as “*an entity, situation, or system which has been selected for analysis*” (Schlesinger, 1979), and constitutes the references against which validations are carried out. When specifying this system of interest it is important to also explicitly define the boundaries in which the modeling is expected to be adequate. The modeling effort itself is separated into the definition of the conceptual model, and its implementation as a computerized model. The conceptual model is an abstract description formed by analysis and observation of the system of interest. In the case of network simulations, the conceptual model takes on the form of a mathematical model describing the dynamics of neurons, the connectivity structure, and other dynamic features of the simulation (e.g., inclusion of neuromodulatory effects). An implementation of the conceptual mathematical model in a computer software or in hardware, on the other hand, results in a computerized, or more concretely for neural simulation, an executable model.

In the context of the formalism laid out by Schlesinger (1979) and refined by Thacker et al. (2004) and others (e.g., Sargent, 2013; Murray-Smith, 2015), validation has a precise definition. Indeed, despite some minor discrepancies, the various definitions of verification and validation agree on the essential aspects. Here we summarize the definitions adapted to neural network modeling and simulation as presented in detail in our companion study (Trensch et al., 2018) in an effort to present a formal terminology for the validation framework developed in this study. The process of ensuring that the executable model is a correct realization of the mathematical model is termed “verification.” In contrast, the comparison of the predictions generated by the computerized model to the system of interest considering its intended domain of applicability is the process called “validation.” Together with the process of “confirmation,” which attributes plausibility to the mathematical model as a useful description of the system of interest, these three attributes form a circle that typically receives multiple iterations consisting of improvements of the mathematical model and its implementation as an executable model. While our companion study (Trensch et al., 2018) investigates primarily the verification step, this study addresses the complementary validation process.

In practice, the conceptual steps are likely to be highly intertwined. In particular, for validation there are two principal scenarios in which a failed validation step impacts the verification. In a first scenario, a validation of the model may lead to an unacceptable discrepancy, which by its nature and appearance, triggers a verification step to detect a previously undetected deficiency of the implementation. In a second scenario, the validation is followed by a further sophistication of the underlying mathematical model. This process of sophistication can be performed either by ignoring the validation outcome, or by explicitly considering it. In the former case, the structure of the mathematical model is evaluated based on modeling the constituent features of the system of interest alone. In the latter case, the model is altered with the explicit aim to improve the validation result, guided by intuition of the scientist on how features and parameters of the mathematical model will influence its output in a simulation, or even supported by a brute–force parameter scan.

This latter type of approach is no longer a true validation step, as it represents a “fitting,” “calibration,” or “optimization” procedure of the model in order to generate a particular desired output behavior. An example of such a procedure is the automatic fitting of single neuron models to experimental data, as performed using tools such as bluepyopt (Van Geit et al., 2016). However, one should consider that, first, as a result of fitting the mathematical model may be altered in ways that are no longer motivated by the underlying system of interest, and second, the fitting is not unbiased in that, by definition, it improves the validation of certain features of the model at the cost of those not included in the fitting procedure. Manipulating a parameter until an observable is within the expected margin of error generally reduces the predictive power of the model. Therefore, validation shall never result in the adaptation of the model the way it is done for fitting. In contrast to validation, fitting parameters within biological reasonable bounds is legit and common practice in a data-driven modeling approach. Even though such calibration and validation seem very similar in practice, they need to be clearly separated. Consequently, models that are calibrated by use of a particular data set require a second data set to perform a rigorous validation test (Thacker et al., 2004).

Since the publication of the depiction of the validation process shown in Figure 1 many derived diagrams have been employed which emphasize additional aspects, for example, the uncertainties in experiment and simulation and their quantification. Other, more complex diagrams point out that model validation is an ongoing and iterative process within a larger workflow of modeling and experimentation (Murray-Smith, 2015). Notable is the explicit inclusion of the validation of experimental data (Sargent, 2013). Both the model building process and the validation rely on experimental data. These data need to be adequate and correct to ensure that the validation is actually meaningful.

### 2.2. Network-Level Validation

There exists a large repertoire of tests and methods to validate a neural network model. The choice depends on the model, its intended use, the nature of the data, and the system of interest. Outside of neuroscience, however, efforts to group validation methods into phases and extract common schemes date back four decades (Forrester and Senge, 1980). These phases include validation on the basis of the model's structure (e.g., model dimensionality and complexity, or the model's behavior in boundary cases as a result of the model simplification), its behavior (e.g., predictive qualities of the executable model, or its robustness under parameter variations or noise), and its response under policy changes (i.e., whether the behavior of the system of interest under change of external policies are reflected by the model, such as when changing the experimental paradigm in a neuroscientific experiment).

In the context of neural network models in neuroscience, one common approach is to start the process of validating the model in an iterative fashion from the level of the smallest elements of the network, for example, the validation of single neuron responses or synapse behavior to experimental data under application of a constant current injection (see e.g., Markram et al., 2015). This single-cell validation is based on the reasoning that when the basic building blocks of a system are validated the resulting system composed of many of the validated building blocks should consequently also perform appropriately. The validation of a larger, or even the entire, system is carried out only once all previous validation tests of the sub-elements have passed with reasonable agreement.

However, the link from the function of the smallest elements to the function of larger composite systems is in general not known, i.e., itself part of the modeling. The difficulty is inherent to multiscale models where emergent properties of a system interact with the dynamics of the constituting elements (Noble, 2006). Nonlinear effects and sensitivity of individual neurons and circuits of connected neurons to parameter changes (Marder and Taylor, 2011) prevent a conclusive prediction of the behavior of the complete model system. Moreover, in models where the individual cells or sub-circuits are simplified and abstracted (e.g., Potjans and Diesmann, 2014), the focus is placed on the question to what extent global features of the dynamics emerge from the network structure as opposed to the details of the elements (e.g., Schmidt et al., 2018). The advantage of simplified neuron models is that their dynamics can be mathematically approximated (for recent examples see Ostojic et al., 2009; Renart et al., 2010; Litwin-Kumar and Doiron, 2012; Schuecker et al., 2015; Bos et al., 2016) enabling a better understanding of the governing mechanisms. Despite their relative simplicity, networks of such model neurons reproduce many dynamical features observed in experimental data (Shadlen and Newsome, 1998; Renart et al., 2010), e.g., the characteristic firing patterns of cortical layers (Potjans and Diesmann, 2014). For such models, the success of single-cell validation is necessarily limited to the single-cell level.

Therefore, we propose network-level validation as a complementary approach that validates the collective dynamics of a network model using the statistical properties of the network spiking activity. Network-level validation is an essential complement to single-cell validation. First, the network dynamics is likely to be a sensitive indicator for critical weaknesses of the model and offers the possibility to detect these early on in the model development process. Second, network-level validation techniques can be applied to abstracted classes of models. Thus, network models with different premises may be compared and validated in a similar manner, including models which lay their emphasis on macroscopic properties of the network. For example, the network dynamics emerging from the interaction of rate neurons can be validated in the same way as spiking neuron based network models using appropriate rate-based validation methods.

### 2.3. Model-to-Model Validation

So far, we considered a scenario in which a model is compared to experimental observations. However, there are circumstances in which a model is the object of reference. This model could be another implementation of the model under scrutiny, an alternative model, or a different simulation run of the same model. In the following, we explore such validation scenarios, which we collectively term “model-to-model” validation.

One possible scenario is the need to demonstrate the equivalence of alternative implementations of the same model. These implementations could, for example, be realized by different simulation engines, for example NEST (RRID:SCR_002963; Gewaltig and Diesmann, 2007), BRIAN (RRID:SCR_002998; Goodman and Brette, 2009), and NEURON (RRID:SCR_005393; Carnevale and Hines, 2006) all having overlapping domains of application. Here, the implementation of a model must take into account the specific features and limitations of a given simulation engine, e.g., the numerical precision. Thus, the choice of a simulator may influence the simulation outcome. Fortunately, there are efforts to overcome the simulator specificity, for example in form of the simulator independent modeling language PyNN (RRID:SCR_002715; Davison et al., 2008). Nevertheless, this approach remains dependent on the degree to which the target simulator adheres to the PyNN model description.

**Figure 2:**
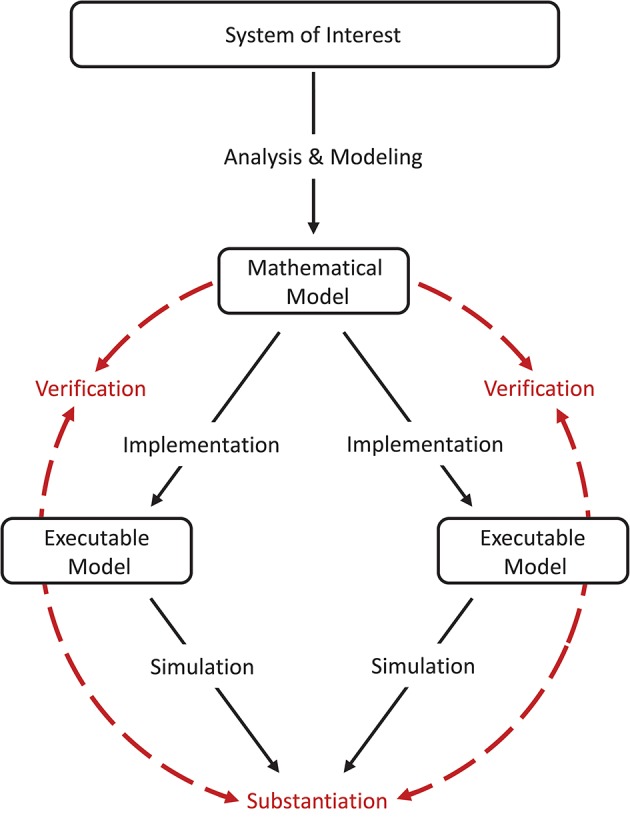
Model verification and substantiation workflow. The workflow shown is an adaption of the verification and validation processes (Figure 1) for the comparison of two executable models (i.e., a model-to-model validation test). The executable models share the same system of interest and the same mathematical model, but differ in the model implementation (e.g., by using different simulation engines). We propose the term “substantiation” instead of “validation” to indicate that this assessment activity cannot evaluate the accuracy of the model with respect to its system of interest. Modeling and simulation activities are indicated by black solid arrows, whereas assessment activities are indicated by red dashed arrows. Figure amended from Trensch et al. (2018).

The comparison between one model and another which is known to be more accurate (e.g., by means of an independent verification process or by validation against experimental data) may also be considered a validation technique in the sense that the latter model is defined as a reference (Martis, 2006). Testing against another model which is already rigorously validated can be described as a “cross-validation.” In the special case where two non-validated implementations based on the same model are used in the model-to-model validation, we are left with an incomplete model assessment process, where there is no direct relation back to the system of interest. Consequently, we use the term “substantiation” instead (Figure 2), in order to not mistake this process for the validation of the model itself, which still requires conventional validation testing including experimental data. Trensch et al. (2018) describes substantiation as “*the process of evaluating and quantifying the level of agreement of two executable models.”* An example of such a situation is the use of validation techniques to disambiguate the effects of implementing a given model using different integration strategies or different simulation engines (van Albada et al., 2018).

Another application of a model-to-model validation is to check for the robustness of a given model with respect to a specific parameter change (see e.g., De Schutter and Bower, 1994). This parameter change may involve a random seed that controls the stochastic input to a model or other model parameters that are based on experimental observations. Such variation of model parameters can assess if a feature of the model behavior robustly emerges from the simulation and is reproducible. The check for robustness is important because experimentally based model parameters are usually observed with a given uncertainty and there are methods to map the influence of this measurement uncertainty to the model output (UncertainPy^12^, Tennøe et al., 2018). For a reasonable sensitivity analysis of the model, however, multiple simulation runs are needed to represent the multidimensional parameter space (Saltelli, 2002; Marino et al., 2008; Zi, 2011; Borgonovo and Plischke, 2016).

Lastly, model-to-model validation is a useful tool in accompanying model development. The flexibility and coverage of a model's dynamics when testing a model against experimental data is often limited due to the scarcity and specificity of available experimental data. Thus, model-to-model testing provides the opportunity to validate the model outcome in a larger space of dynamical regimes not necessarily covered by available data.

The statistical methods presented in this study are generally suitable for model assessment, i.e., model validation against experimental data and model-to-model validation, including substantiation scenarios. To emphasize this generality, the term validation is used throughout the entire manuscript, even if the worked example considers substantiation.

## 3. Methods

### 3.1. Methods for Network-Level Validation

For network validation one usually cannot expect a spike-to-spike equivalence between the simulated spiking activity and the experimental data or between two models. Even for different implementations of the same model, the computation depends on the capabilities and limitations of the computer hardware and the exact details of the computer environment (Glatard et al., 2015). Therefore, the simulation outcomes must be compared statistically in order to quantify the level of similarity. In the following we outline a number of measures of increasing complexity that capture a broad range of network activity dynamics.

Mono- and multivariate measures can, in a sense, be regarded as forming a hierarchical order. Monovariate statistics consider only the single unit activity, irrespective of other units' behavior, while multivariate statistics consider how the pairwise or higher-order activity of units is coordinated within the system. Nevertheless, it should be noted that this conceptual hierarchy does not imply a hierarchy of failure, i.e., a correspondence on the highest order does not automatically imply correspondence of lower order measures. Therefore, it is imperative to independently evaluate each statistical property.

#### 3.1.1. Monovariate Measures

We characterize activity of single neurons in the network using the distributions of several monovariate measures. The level of network activity can be estimated by the average firing rate

(1) $$\begin{array}{l} FR=n_{sp}/T, \end{array}$$

where *n*_*sp*_ denotes the number of spikes during an observation interval of length *T*. The inter-spike intervals are defined by

(2) $$\begin{array}{l} {ISI}_{i}=t_{i+1}-t_{i}, \end{array}$$

where *t*_*i*_ denote the ordered spike times of a neuron. The distribution of *ISI*_*i*_ is used to characterize the temporal structure of the single spike trains. A measure particularly suited to analyze the regularity of the spike intervals is the local coefficient of variation

(3) $$\begin{array}{l} LV=\frac{1}{n-1}\overset{n-1}{\underset{i=1}{\sum }}\frac{3{\left(t_{i}-t_{i+1}\right)}^{2}}{{\left(t_{i}+t_{i+1}\right)}^{2}}, \end{array}$$

which is equal to 1 for a Poisson process (Shinomoto et al., 2003).

#### 3.1.2. Bivariate Measures

For pairwise statistics we analyze the cross-correlation function

(4) $$\begin{array}{l} R_{xy}\left(\tau \right)=\langle x\left(t\right)y\left(t+\tau \right)\rangle =\frac{1}{N}\overset{N}{\underset{t=1}{\sum }}x\left(t\right)y\left(t+\tau \right)\;, \end{array}$$

where 〈·〉 denotes the temporal average (Tetzlaff and Diesmann, 2010). It quantifies correlations between spike counts of two binned spike trains, *x*(*t*) and *y*(*t*), for a range of lags τ given *N* bins. Subtracting the average spike counts μ_*x*_ = 〈*x*(*t*)〉 and μ_*y*_ = 〈*y*(*t*)〉 yields the covariance function

(5) $$\begin{array}{l} C_{xy}\left(\tau \right)=\langle \left(x\left(t\right)-\mu_{x}\right)\left(y\left(t+\tau \right)-\mu_{y}\right)\rangle =R_{xy}\left(\tau \right)-\mu_{x}\mu_{y}\;. \end{array}$$

Normalizing the covariance function by the standard deviations $\sigma_{x}=\sqrt{C_{xx}\left(\tau =0\right)}$ of the processes, one obtains the cross-correlation coefficient function

(6) $$\begin{array}{l} \rho_{xy}\left(\tau \right)=\frac{C_{xy}\left(\tau \right)}{\sigma_{x}\sigma_{y}}\;. \end{array}$$

The Pearson correlation coefficient is given by ρ_*xy*_(τ = 0) (Perkel et al., 1967). The matrix of correlation coefficients, *C*, evaluates the non-delayed (i.e., zero-lag) correlation of spikes. The activity on different scales can be analyzed applying different bin sizes. Here we use binned spike trains on a fine temporal scale (Pearson correlations denoted by CC, using a bin width of 2 ms) and on a coarse scale (Pearson correlations denoted by RC, using a bin width of 100 ms). The correlations on coarser scales are often referred to as rate correlation. In particular, RC is able to capture characteristic population-wide fluctuations of network activity that are often observed on the associated temporal scales (see e.g., the stripy asynchronous irregular state in Voges and Perrinet, 2012).

Since the particular model used as an example in the present study was originally conceived to exhibit a spatiotemporal arrangement of the spiking activity (polychronous groups), we analyze in addition potential delayed correlations by considering the cross-correlation coefficient function ρ_*xy*_(τ). We select a bin width of 2 ms and calculate the sum of the cross-correlation coefficient function for lags up to 100 ms, corresponding to an interval of [−Δ; Δ] bins around 0 with Δ = 50:

(7) $$\begin{array}{l} P_{xy}=\overset{\Delta }{\underset{\tau =-\Delta }{\sum }}\rho_{xy}\left(\tau \right) \end{array}$$

in order to quantify the fine temporal correlation including potential lagged correlations.

#### 3.1.3. Correlation Structure

Eigenvectors of the correlation matrix capture the correlation structure of network activity (Friston et al., 1993; Peyrache et al., 2010). Consider the eigendecomposition of the symmetric, zero-lag correlation matrix according to

(8) $$\begin{array}{l} C{\text{v}}_{\text{i}}=\lambda_{i}{\text{v}}_{\text{i}}, \end{array}$$

where λ_*i*_ are eigenvalues and **v_i_** are eigenvectors. Due to the symmetry of the real valued matrix *C* it follows that λ_*i*_ ≥ 0 and eigenvectors **v**_*i*_ are real and orthogonal to each other. A large eigenvalue corresponds to an intra-correlated group of neurons, whose activity explains a large amount of variance in the system, and relates to dominant features in the correlation structure. The loadings of the corresponding eigenvector **v**_*i*_ identify the neurons constituting such groups. Consequently, a suitable sorting algorithm, for example hierarchical clustering, exposes intra-correlated groups as block like features of the correlation matrix. Here, we use the scipy (RRID:SCR_008058; v1.0.0) implementation scipy.cluster.hierarchy.linkage() with method= “ward” and otherwise default settings.

To quantify to which degree the correlation structure of two simulation outcomes (1 and 2) is similar, one may flatten the upper triangular matrices of the correlation matrices *C*_1_ and *C*_2_ into vectors **c**_1_ and **c**_2_, respectively. This omits duplicate entries due to symmetry and the unity auto-correlation on the diagonal. The normalized scalar product

(9) $$\begin{array}{l} 0\leq \frac{\left|{\text{c}}_{1}\cdot {\text{c}}_{2}\right|}{∥{\text{c}}_{1}∥∥{\text{c}}_{2}∥}\leq 1 \end{array}$$

then constitutes a measure of similarity. A value of 1 denotes two identical vectors and a value of 0 two perpendicular vectors. The order of pairwise correlation coefficients in the two vectors **c**_1_ and **c**_2_ needs to be identical, i.e., the similarity measure is sensitive to the labeling of the neurons. Therefore, it should only be applied to compare two network simulations of the same neuron population. Accordingly, reordering the neuron population of one network statistically decreases the similarity measure of any existing structured correlation matrices while preserving the value for non-structured, e.g., homogeneous, correlation matrices. As a test statistic, the distribution of the normalized scalar product is not known and depends on the distribution of cross-correlation coefficients in **c**_1_ and **c**_2_. The significance of the similarity measure is therefore estimated by means of surrogate data. The associated null distribution is computed by randomly shuffling the neuron order of one network 10, 000 times.

#### 3.1.4. Spatiotemporal Patterns

The evaluation of the correlation structure presented so far considers only pairwise measures. Nevertheless the spiking activity of complex networks may include higher-order interactions. Several methods for the detection of higher-order correlation have been developed in recent years (for a review see Quaglio et al., 2018) that do not make any specific assumption about the underlying connectivity and are thus well suited as statistical measures for model validation. Here, we focus on the SPADE (Spike Pattern Detection and Evaluation) method (Torre et al., 2013; Quaglio et al., 2017). SPADE is a statistical method designed to detect spatiotemporal spike patterns, i.e., temporally precise spike sequences, including synchronous spiking activity. The method is composed of two main steps: (a) using Frequent Itemset Mining to detect repeated spike sequences in parallel spike trains, and (b) selecting the sequences that occur often enough to be significant with respect to the null hypothesis of independent firing. The features of the patterns (neurons forming the sequences, number and time of occurrences, lags between the spikes forming the sequence, statistical significance of the pattern) characterize the network activity in terms of higher-order statistics.

#### 3.1.5. Statistical Comparison of Distributions

Consider two sample distributions with means μ_*i*_ and standard deviations σ_*i*_. Here, such sample distributions represent the neuron-wise or pairwise evaluation of one of the measures described above. According to Hedges (1981), the effect size

(10) $$\begin{array}{l} d=\frac{\mu_{1}-\mu_{2}}{\sigma }, \end{array}$$

characterizes the difference of the mean values where

(11) $$\begin{array}{l} \sigma =\sqrt{\frac{\left(n_{1}-1\right)\sigma_{1}^{2}+\left(n_{2}-1\right)\sigma_{2}^{2}}{\left(n_{1}+n_{2}-2\right)}} \end{array}$$

is the pooled standard deviation and the *n*_*i*_ specify the number of samples entering each distribution. In the case of equal sample sizes the definition is equivalent to Cohen (1988, p. 67). In case of multiple simulation runs, we calculate the average effect size of the respective measures. This is possible because the simulations are independent and there is no systematic trend of the measures for the evolving network states. Calculating the effect size assumes that both distributions are Gaussian. Even though this assumption is not fulfilled for every measure, we calculate the effect size as a simple quantification of the difference between the non-normal distributions. Note, that for non-normal distributions a small effect size does not necessarily indicate similarity because there might still be a mismatch in the shape of the distribution. In these cases additional tests are needed to give a more complete evaluation. Candidates are the scalar product measure to compare correlation structures, and statistical hypothesis tests.

The present work employs hypothesis tests to assess the equality of the means (two-sample Student's *t*-test) and the equality of the distributions (Kolmogorov-Smirnov test, Mann-Whitney *U* test). This quantifies the discrepancy in the results by a *p*-value. The two-sample *t*-test is only applicable to normally distributed data, while the latter two tests are non-parametric and thereby applicable to any form of distribution. The Kolmogorov-Smirnov test computes the supremum of the difference of the two cumulative distribution functions, while the Mann-Whitney *U* test compares the rank sums of the jointly sorted samples. In general, when applying hypothesis tests the interpretation of the *p*-values as a similarity assessment must also take into account potential biases and dependencies, e.g., on the sample size, and the simulation time (Cohen, 1994).

### 3.2. Implementation of Validation Tests in a Modular Framework

Rigorous validation testing requires that test results are not affected by details of the actual testing procedure. This translates to performing the extraction of test statistics and its evaluation with the exact same methods for both data sources entering the test. In a more complex scenario, this also includes finding an appropriate mapping between the data sources, for instance when comparing a large-scale simulation of spiking activity to experimental data taken from few electrodes only. Ultimately, validation methodologies should be standardized within the neuroscientific community to ensure consistency of the validation scores across different validation cycles of related models or data sets. The starting point for drafting a common base for validation testing is the formalization of the validation workflow for the individual research domains. For network-level validation of spiking activity data we created this formalization as the open-source Python module NetworkUnit^13^ (RRID:SCR_016543). All quantitative comparisons of statistical measures of this study are carried out in this framework and the workflow to reproduce the findings of this study using NetworkUnit is available online as a Jupyter notebook^14^.

**Figure 3:**
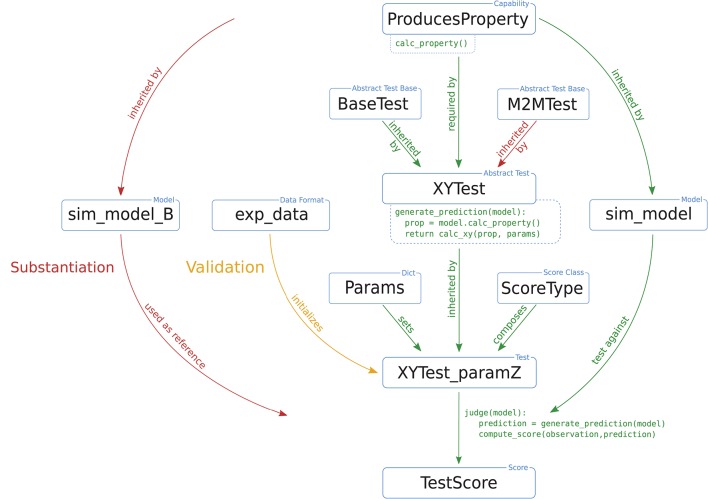
Illustration of a typical test design within NetworkUnit. The blue boxes indicate the components of the implementation of the validation test, i.e., classes, class instances, data sets, and parameters. The relation between the boxes are indicated by annotated arrows. The basic functionality is shown by green arrows. The difference in the test design for comparing against experimental data (validation) and another simulation (substantiation) is indicated by yellow and red arrows, respectively. The relevant functionality of some components for the computation of test score is indicated by pseudo-code. The capability class ProducesProperty contains the function calc_property(). The test XYTest has a function generate_prediction() which makes use of this capability, inherited by the model class, to generate a model prediction. The initialized test instance XYTest_paramZ makes use of its judge() function to evaluate this model prediction and compute the score TestScore. The XYTest can inherit from multiple abstract test classes (BaseTest), which is for example used with the M2MTest to add the functionality of evaluating multiple model classes. To make the test executable it has to be linked to a ScoreType and all free parameters need to be set (by a Params dict) to ensure a reproducible result.

NetworkUnit focuses on the statistical comparison of measures characterizing spiking neural network models. It is based on the Python package SciUnit (RRID:SCR_014528; Omar et al., 2014), which provides a generic basis for the testing of models, employing similar concepts to those of unit testing in software engineering. SciUnit consists of three base classes for models, tests, and scores. The model class defines the model to be validated and, if needed, handles its execution. The test defines which measure, or feature, is to be extracted from the model, and defines against which experimental data the model is to be validated. Finally, the score defines the validation method to be applied and quantifies the result of the validation cycle. Models and tests are connected via their capabilities, e.g., a definition of what types of data output a model provides, and what type of data input the test requires to extract its measure. Figure 3 schematically depicts the interplay of these components and the class hierarchy for the cases of validation of a model against experimental data or substantiation against another model.

For the analysis presented in this paper, the components in Figure 3 can be understood as follows: the basic underlying capability is the class ProducesSpikeTrains as all analyzed measures are based on the spike times. The SpiNNaker model is implemented as the sim_model that is to be validated. It could either be validated against experimental data (exp_data), or substantiated against another instance of the model (sim_model_B), e.g., the original implementation as illustrated in our worked example. The test statistics we use in XYTest are the distributions of the measures presented in Section 3.1, e.g., firing rate or correlation coefficient. All these tests involve the comparison of distributions, so they are derived from a corresponding BaseTest (and potentially additional base tests). Some statistics, e.g., the correlation coefficient, depend on additional parameters (controlled by Params) such as the binsize. The ScoreType in our case are statistical hypothesis tests or the effect size.

The test instance uses spike trains from the model and the experimental data or, as in our case, from the reference model implementation to generate a “prediction” and an “observation,” respectively. The calculation of features on activity data is performed using the Electrophysiology Analysis Toolkit^15^ (Elephant, RRID:SCR_003833). Both observation and prediction are passed on to the score class, which evaluates their statistical congruence, e.g., in form of a two-sample *t*-test. Finally, the judge function of the test instance returns the results, for example the *p*-value of the statistical hypothesis test. This design formalizes the generation of the results and makes them reproducible. The modular design of model and test classes enables the reuse of existing tests which facilitates the comparison of results of different models.

In practice, performing a single test for validating a model does not sufficiently capture the model behavior to comprehensively quantify it and document the model's scientific applicability. Thus, a whole range of validation tests is usually performed, which may in some cases differ only in details or may depend on a parameter. Instead of rewriting the test definition each time, it is more feasible to make use of class-based inheritance as indicated in Figure 3 (BaseTest→XYTest→XYTest_paramZ). All specific tests derive from the sciunit.Test base class. They add and overwrite the required functionality, as for example generating the prediction by calculating the correlation coefficients from spike trains. Because there may be a lot of different tests making use of correlation coefficients (for example, calculating correlations on different time scales), it is recommended to implement first an abstract generic test class to handle correlations. This abstract test class cannot be accessed explicitly by a user but only acts as a parent class for the actual executable test class, which, e.g., implements the test for a specific choice of the bin size. This class-based inheritance guarantees that all tests build on the same implementation and workflow.

In this study we concentrate on model-to-model validation. In this scenario, the test instance compares the prediction of two model instances and accordingly needs to accept two model instances as input. For that scenario, SciUnit provides the test class TestM2M, in which the experimental data (exp_data) in Figure 3 are replaced by a second model class (sim_model_B).

### 3.3. Substantiation of the Izhikevich Polychronization Model

In a companion study, Trensch et al. (2018) demonstrate a rigorous model substantiation workflow. In a first step, the authors replicate a published minimal spiking network model, capable of exhibiting the development of polychronous groups of spiking neurons (Izhikevich, 2006), referred to in the following as the “polychronization model.” In a further step, the study details the iterative processes of implementation, verification, and substantiation of the original implementation of the polychronization model against a reproduction on the SpiNNaker neuromorphic system. Trensch et al. focus on the refinement of the implementations and their verification, i.e., the source code verification and calculation verification, and address the question of the degree of numerical precision required on neuromorphic systems. This is complemented by this study focusing on the details of the corresponding substantiation process, the testing for equivalence of statistical features of the collective dynamics in five selected network states. This section summarizes the polychronization model description, the simulation setup, and the model substantiation procedure of Trensch et al. (2018).

#### 3.3.1. Polychronization Model

We chose the polychronization model (Izhikevich, 2006) to demonstrate a rigorous model substantiation process. The choice was motivated by a number of non-standard features in its conceptual and implementation choices that make it an illustrative example for the source code and calculation verification process conducted in a complementary study (Trensch et al., 2018) and, particularly, for an reproduction on the SpiNNaker (Furber et al., 2013) neuromorphic system. The model exposes essential aspects in the formalization and simulation of neural networks as it produces a rich repertoire of network dynamics. Note that we do not evaluate the emergence of polychronous groups, as this turns out to be rather sensitive to details of the implementation choices. For a comprehensive investigation of this aspect, see Pauli et al. (2018). The original model is implemented in the C programming language and is available for download from the website of the author^16^.

The polychronization model consists of 1,000 neurons with four times more excitatory than inhibitory neurons. Each neuron is described by the model specified in Izhikevich (2003). In accordance with the definitions by Izhikevich, excitatory neurons are parameterized to exhibit regular spiking, and inhibitory neurons to show fast spiking behavior. The neurons are connected randomly with a fixed out-degree of 100, where inhibitory neurons only form connections to the excitatory population. Each excitatory connection is assigned a fixed delay drawn from a discrete uniform distribution between 1 and 20 ms in intervals of 1 ms and all inhibitory connections are assigned a delay of 1 ms. Synaptic weights are initialized with an initial value of 6 for excitatory and −5 for inhibitory connections. The original model uses dimensionless variables, however, currents can be interpreted in units of pA. The network is driven by random input realized by an external current pulse of 20 pA injected into one randomly chosen neuron in each time step. The simulation time step is 1 ms, within which multiple intermediate steps are calculated, depending on the implementation (Trensch et al., 2018). The stimulated spiking activity in the network modifies the connection weights according to a spike-timing-dependent plasticity (STDP) rule. Synaptic weight changes are buffered for one biological second and then the weight matrix is updated for all plastic synapses simultaneously. We leave out a detailed description of the implementation of plasticity here because it is not of relevance for the remainder of the study as it considers only the dynamics after freezing the learned connectivity matrix, and refer to Pauli et al. (2018).

#### 3.3.2. Simulation Setup

**Figure 4:**
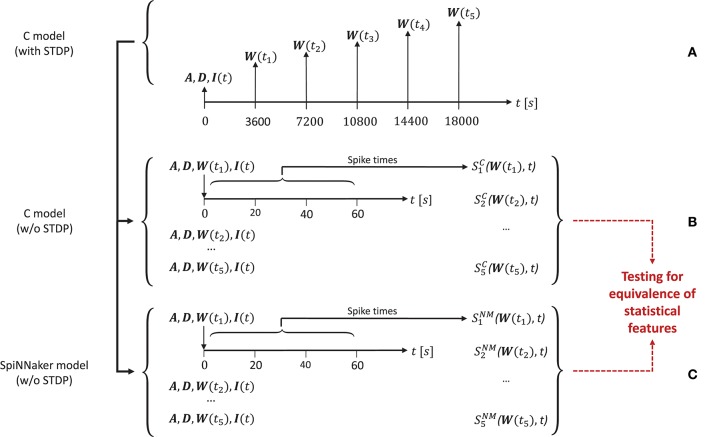
Design of the simulation setup. The time line is annotated by the variables saved or loaded at specific time points of the simulation for the three types of simulations used in the substantiation scenario. **(A)** Generation of the five initial network states used to simulate data. At the start (*t* = 0*s*) of running the C implementation of the polychronization model (with STDP) the connectivity matrix ***A*** and delay matrix ***D*** are saved. At the following times *t*_*i*_, the weight matrix ***W***(*t*_*i*_) is saved. The random input stimulus to the network ***I***(*t*) is recorded for the duration of the simulation. **(B)** Generation of data from the five simulations of the C implementation (without STDP) for use in the validation tests based on the random input ***I***(*t*) and the five sets of initial conditions (***A***, ***D***, ***W***(*t*_*i*_)) recorded in **(A)**, respectively. The network spiking activity $S_{i}^{\text{C}}\left(\text{W}\left(t_{i}\right),t\right)$ is recorded for 60 s. **(C)** Identical setup as in **(B)**, but for the SpiNNaker implementation without STDP, where $S_{i}^{\text{NM}}\left(\text{W}\left(t_{i}\right),t\right)$ denotes the simulation result. The data from **(B,C)** are subject to validation testing based on their statistical features (red dotted lines). Figure amended from Trensch et al. (2018).

Trensch et al. (2018) consider for the validation task the dynamics of the original C implementation of the polychronization model in five arbitrarily selected network states. Figure 4 illustrates the setup of the simulation. Analyzing five network states within one simulation process instead of the outcome of multiple different simulations with different random seeds is motivated by the findings of Pauli et al. (2018) who show that the model may converge into two distinctly different activity states. By analyzing the sample activity at different training times within one simulation this ambiguity problem for the analysis is bypassed. In order to generate the network activity data for the statistical analysis and to save the network states, the authors perform the following three steps:

1. Execute the C implementation with STDP for 5 h of biological time. During this simulation run, save the network state at five points in time *t*_*i*_, *i* = (1, 2, …, 5) after 1, 2, 3, 4, and 5 h. The network state is defined by the weight matrix ***W***(*t*_*i*_) containing the current strength of each synapse, the connectivity matrix ***A***, and the delay matrix ***D***. Additionally, record the first 60s of the random series of neurons to which the external stimulus is applied (***I***(*t*), Figure 4A).
2. Switch off STDP in the C implementation. Re-initialize the network model with ***A***, ***D***, ***I***, and the respective ***W***(*t*_*i*_) for the five simulation runs *i* = (1, 2, …, 5). In each run record the network spiking data $S_{i}^{\text{C}}$ over 60 s (illustrated in Figure 4B).
3. Repeat step (2) with the implementation on the SpiNNaker neuromorphic system (NM) of the polychronization model to obtain the spiking data $S_{i}^{\text{NM}}$.

The spiking data $S_{i}^{\text{C}}$ and $S_{i}^{\text{NM}}$ are then subject to the statistical analysis and comparison described in detail in the present work. Note that for the sake of simplicity only the excitatory population is considered in the following validation, yet the results for the inhibitory population do not differ qualitatively.

#### 3.3.3. Substantiation Workflow

The complementary study (Trensch et al., 2018), which details the activities of implementation, verification and validation conducted in the course of the substantiation process, presents three iterations of the entire workflow. In the following, we summarize the actions taken in these iterations. As each of the iterations demonstrates a different aspect of validation testing, the present study refers to the corresponding iteration where suitable.

First, the original C implementation of the polychronization model (Izhikevich, 2006) underwent a source code verification, inspection and refactoring task, while paying attention to preserving bit identity, i.e., bit-wise replicability, of the simulation outcome. A reproduction of the polychronization model was implemented on the SpiNNaker neuromorphic system using the Izhikevich neuron model implementation provided by the SpiNNaker software stack, using the Explicit Solver Reduction (ESR) implementation of the dynamics described in Hopkins and Furber (2015). The substantiation of a choice of statistical features exposed discrepancies. This led the authors to the definition of verification tasks, in terms of calculation verification, to verify the accuracy of the numerical algorithms and computations.

The second iteration carried out these verification activities. As a result, the ODE solver implementation for both, the SpiNNaker and the C model, was replaced by a semi-implicit fixed-step size forward Euler scheme. Additionally, the revised implementations include a precise threshold detection, and for some critical calculations an optimized fixed-point representation for improving the numerical precision of computations.

The third iteration is concerned with a shift that was observed in the LVs but not in the other monovariate measures such as the firing rate. The formalized workflow of verification and validation uncovered this shift to be caused by an implementation issue leading to a small systematic lag in spike timing. Each iteration thus constitutes a refinement of the implementation step with a subsequent verification assessment and a substantiation (utilizing NetworkUnit) as depicted in Figure 2. A short summary of the specific changes in each iteration is depicted in Table 1. The model source codes, simulation scripts and the codes used in the verification activities, developed in our companion study, are available on GitHub^17^.

**Table 1:** Summary of the development steps of the model implementations.

	**C model**	**SpiNNaker model**
**Iteration I**	Uses a semi-implicit fixed-step size forward Euler ODE-solver with step size 1 ms	(i) Uses the SpiNNaker Explicit Solver Reduction (ESR) implementation of the Izhikevich neuron model
		(ii) Uses Izhikevich's algorithm for the neural dynamics (iii) Uses a more exact fixed-step size forward Euler ODE-solver with step size 1 ms.
**Iteration II**	Uses a 1/16 ms step size and a more precise detection of threshold crossing	Uses a 1/16 ms step size and a more precise detection of threshold crossing Applies fixed-point conversion for critical calculations
**Iteration III**	Remains unchanged	Resolves an implementation issue with the threshold detection

*The iterative development of the simulation codes is based on a replication of Izhikevich's original implementation. The steps (ii) and (iii) represent incremental improvements in between iterations I and II. ODE, ordinary differential equation*.

## 4. Results

In this section we present the results of the various validation tests of the SpiNNaker implementation against the C simulation of the polychronization model. Pauli et al. (2018) expose that the model dynamics is sensitive to small changes in model parameters and numerics. Accordingly, we do not expect a spike-to-spike equivalence between the SpiNNaker neuromorphic system, which makes use of 32-bit fixed-point numerics, and the C implementation, employing floating-point numerics. Hence, any comparison needs to rest on statistical measures. Following the results of the various validation tests of the SpiNNaker implementation against the C simulation, in Section 4.1 we show that the application of validation tests during model development and implementation quantifies and guides the progress. Section 4.2 demonstrates the importance of incorporating multiple measures in the validation of network activity, since the agreement of a higher order statistical measure does not entail the agreement of measures of lower order. As the last step of the validation process Section 4.3 uses a selection of test measures and scores to comprehensively validate the SpiNNaker model implementation against the C implementation.

### 4.1. Comparison of Network Activity During Implementation

**Figure 5:**
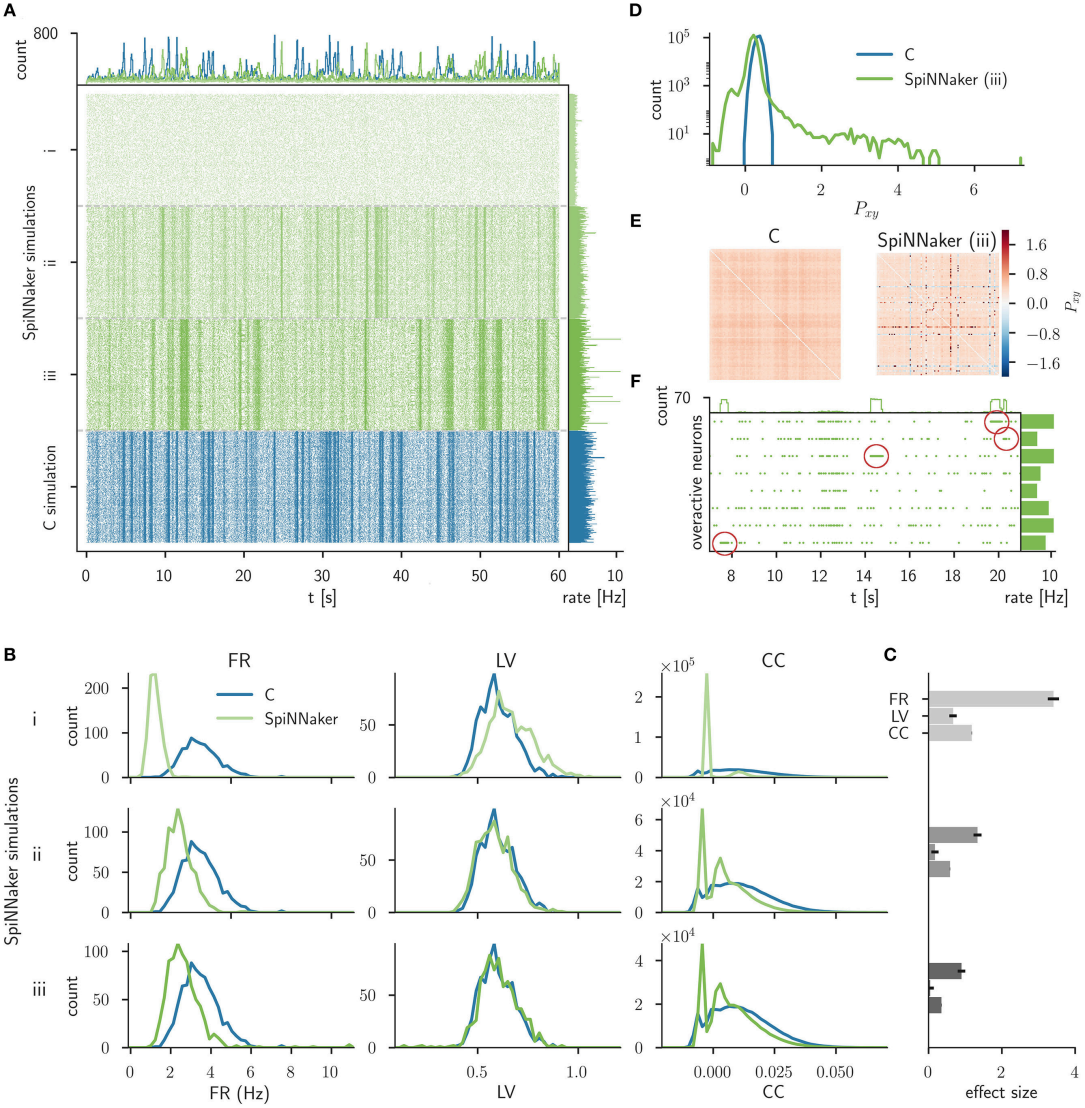
Comparison of the C simulation with simulations of three consecutive stages of the SpiNNaker implementation. **(A)** Raster plot of the spiking network activity (800 excitatory neurons) of the C simulation (bottom, blue) and three stages of the SpiNNaker implementation; i, ii, and iii (top, shades of green). The top and right histograms show the population spike counts in 60 ms bins and the mean firing rates, respectively. **(B)** Distributions of firing rates (FR, left), local coefficients of variation (LV, middle), and correlation coefficients (CC, right) for the C and SpiNNaker simulations. Each row (subsequent implementation steps: i, ii, iii) represents a specific SpiNNaker simulation (green) that differs in the underlying neuron model implementation. Data shown for the C simulation (blue) are identical in the three rows. **(C)** The difference between the distributions is quantified by the effect size with error bars indicating the 95% confidence interval. In step iii the effect sizes for the FR, LV, and CC measure are 0.90, 0.05, and 0.36, respectively. **(D)** Distributions of the sum of the cross-correlation coefficient (*P*_*xy*_, Equation 7) in logarithmic representation for C and SpiNNaker (implementation step iii). **(E)** Color coded correlation matrices for the sum of the cross-correlation coefficient in implementation step iii. The symmetric matrices display results for the subset of 100 excitatory neurons with highest spike rates in the SpiNNaker simulation. **(F)** Raster plot of 8 overactive neurons in the SpiNNaker simulation (implementation step iii) showing episodes of 1 kHz spiking (emphasized by red markers). The top and right histograms show the population spike counts in 60 ms bins and the mean firing rates for the entire recording, respectively.

The modeler already benefits from the use of quantitative statistical comparisons for model validation during the iterative process of model implementation. Based on our example, we demonstrate this by the improvements of the implementation on SpiNNaker obtained in three iterative steps denoted by i–iii in Figure 5 (see also Table 1). The results shown are taken from 60 s of simulated data starting from the network state after 5 h of biological time.

Figure 5A displays the spiking data of the C implementation (corresponding to iteration I in Trensch et al., 2018) compared to the three consecutive steps of the SpiNNaker implementation. Step i denotes the initial SpiNNaker implementation using an Explicit Solver Reduction (ESR) algorithm for the Izhikevich neuron dynamics (see iteration I in Trensch et al., 2018). In step ii this algorithm is replaced by a reimplementation of the neuron dynamics described in Izhikevich (2006). Step iii improves this algorithm, by applying a fixed step size forward Euler method (see iteration II in Trensch et al., 2018). Step i does not exhibit the strong fluctuation of the population activity (visible as vertical stripes in the raster plot) that are present in the C simulation. The following SpiNNaker simulation steps ii and iii, in contrast, do exhibit these fluctuations. As expected, none of the SpiNNaker simulations show a spike-to-spike equivalence with the C implementation.

In order to assess the statistical agreement between the C and SpiNNaker simulations during implementation development, we compare the distributions of FRs, LVs, and pairwise CCs using the effect size defined in Section 3.1.5. The results are shown in Figure 5B for the 9 comparisons (3 steps, 3 measures). Visually, the agreement between the C and the SpiNNaker simulations improves with each step of the SpiNNaker implementation. This is also quantitatively confirmed by the effect size displayed in Figure 5C. This information guides the modeler in assessing the model improvement in the iteration steps. The effect size declines with each iteration step consistently for all measures. However, despite the good visual agreement of the raster plots for the final step, the discrepancy in the distributions of firing rates is still considerable. There remains also a shape mismatch between the distributions of CCs (step iii, Figure 5B).

The distribution of the sum of the cross-correlation coefficient of the SpiNNaker simulation (step iii, Figure 5D) is much broader than the distribution obtained from the C simulation and also shows a much larger tail, while the distribution for the C simulations is close to a Gaussian. The corresponding correlation matrix (Figure 5E) for SpiNNaker reveals that the largest values as well as the smallest values causing the deviation are arranged in horizontal and vertical lines. The correlation matrix for the C simulation, on the other hand, does not show similar outliers. The line structure uncovers individual neurons that are highly correlated or anti-correlated (within a ±100 ms delay window) to a large number of other neurons. Further investigation reveals 8 particular neurons, that in the following we refer to as overactive neurons. These overactive neurons not only cause the long tail in the distribution of integral correlations *P*, but also exhibit larger firing rates than the rest of the population. This suspicious behavior motivates a closer look at their spiking activity revealing occasional episodes with firing rates of 1 kHz for several hundred milliseconds (see Figure 5F for an illustration of such episodes). Subsequent analysis and review of the source code determines an implementation issue of the neural dynamics as the origin of the problem. The episodes in question are triggered by an overflow of a fixed-point variable in the calculation of the membrane potential. Thus, the validation process reveals a mismatch in the dynamics that provides valuable information to guide a subsequent verification step.

### 4.2. Differential Effects on Statistical Measures

Next, we investigate the statistical properties of spiking activity for the SpiNNaker implementation resulting from the next iteration step that addresses the overflow discussed above. Briefly, the refinements of the SpiNNaker and the C code are the employment of an improved forward Euler ODE solver, a precise detection of threshold crossings, and a more accurate fixed-point representation on SpiNNaker (for details, see Table 1 and iteration II in Trensch et al., 2018).

**Figure 6:**
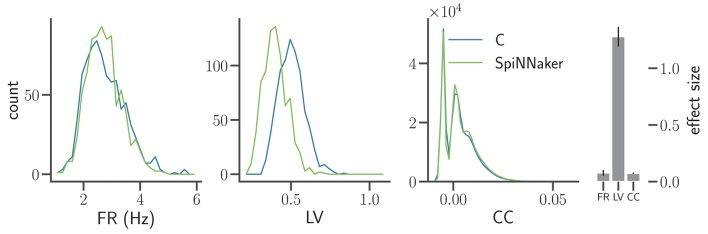
Comparison of statistical measures after model refinement. The panels show from left to right the distributions of FR, LV, and CC of the C and SpiNNaker simulation after the first refinement of the implementations by Trensch et al. (2018) for the network state after *t*_5_ = 5 h (same display as in Figure 5B). The histogram on the right visualizes the effect size in the three statistical measures (mean and standard deviation across all five network states *t*_1_, *t*_2_, …, *t*_5_). The numerical values are FR: 0.077 ± 0.025, LV: 1.28 ± 0.086, and CC: 0.074 ± 0.006 respectively.

In Figure 6 the distributions of mean firing rates and correlation coefficients show a good agreement in terms of effect sizes and an overall better visual agreement of the shapes of the distributions (Figure 5B, bottom row). The LV distributions, however, exhibit a clear shift toward lower values not present in the previous iteration, reflected by an increased mean effect size. The spiking activity in the SpiNNaker simulations is therefore considerably more regular despite similar mean firing rates and pairwise correlations as the C simulation. Thus, Figure 6 illustrates a situation where the refinement of an implementation improves two statistical measures while it worsens a third. The implementation process needs to be accompanied by the simultaneous consideration of multiple statistics.

### 4.3. Comprehensive Assessment and Higher-Order Collective Properties

The refinement of the last iteration is the correction of the threshold detection algorithm of the SpiNNaker implementation, while the C simulation remains unchanged (for details, see Table 1 and iteration III in Trensch et al., 2018). At this point, the effect sizes of the statistical measures decreased substantially, suggesting the inclusion of further measures of the collective properties of the system into the validation. In this way we obtain an impression of how far the present measures constrain the dynamics of the system and to what extent higher-order measures of interest for the experimentalist are preserved.

**Figure 7:**
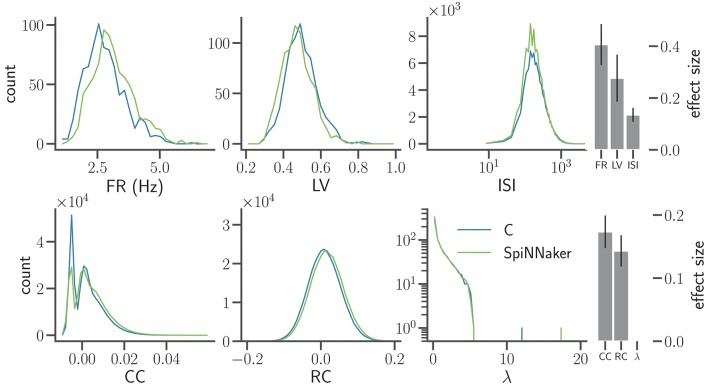
Distributions of characteristic measures of network activity simulated with C and SpiNNaker after the final step of refinement. The top row shows single neuron statistics FR, LV, and the ISIs (same display and data specification as in Figure 5B). The histograms of ISIs are displayed using semi-logarithmic scaling. The bottom row shows pairwise statistics and network properties, namely the CC using 2 ms bins, the rate correlation (RC) using 100 ms bins, and the eigenvalues (λ) of the RC matrices displayed on the vertical axis using a logarithmic scaling. Right: effect size using the same display as in Figure 6. The effect sizes for the tested measures are FR: 0.41 ± 0.08, LV: 0.28 ± 0.09, ISI: 0.14 ± 0.03, CC: 0.17 ± 0.03, RC: 0.14 ± 0.02, and λ: < 8 × 10^−17^, respectively.

Figure 7 shows the three distributions considered in previous iterations (FR, LV, and CC; cf. Figures 5B, 6) and in addition the distributions of the ISIs, the RC, and the eigenvalues (λ) of the rate correlation matrices. According to the interpretation of Cohen (1988), the comparisons of all six measures exhibit effect sizes of small to medium size.

Compared to the previous iteration (Section 4.2), the LV of the SpiNNaker implementation better matches the C implementation. The firing rates, however, now show a small but systematic shift to larger rates compared to the C simulation. Despite a slight increase in the effect size for firing rates and correlation coefficients, the overall agreement in terms of the effect sizes improves due to the improved match of the LV distributions. The distributions of ISIs appear log-normal and are well matched. The higher peak in the distribution for the SpiNNaker simulation results from the increased firing rates in the SpiNNaker simulation.

The SpiNNaker simulations also show a small shift to larger RC. For the C and SpiNNaker simulations the corresponding distributions of eigenvalues (λ) of the rate correlation matrices are similar. Both distributions have a single eigenvalue that is considerably larger than the rest. Therefore, one single mode explains a large part of the total variance of the population activity. This largest eigenvalue, however, is considerably larger for the SpiNNaker simulation. This indicates that the intermittent increases of population activity observed in SpiNNaker are larger in terms of amplitude compared to the C simulation (see e.g., the oscillations described by Bos et al., 2016).

We test for equivalent sample distributions of all six measures shown in Figure 7 using the non-parametric Kolmogorov-Smirnov test and the Mann-Whitney *U* test for all 5 network states. We also apply the parametric Student's *t*-test to those measures which are approximately Gaussian distributed (FR, LV, RC, log(ISI)). All tests reject their null hypotheses with *p*-values clearly below a 5% significance level (without correction for multiple comparisons). The only exception are the eigenvalue distributions, which yield *p*-values between 0.17 and 0.96 for the 5 network states. In conclusion, all but the eigenvalue distributions are statistically different.

**Figure 8:**
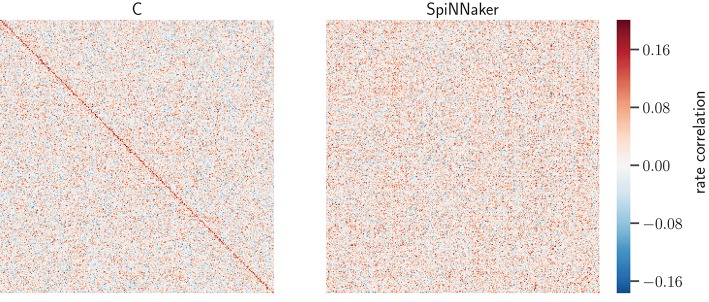
Rate-correlation matrices for the C and SpiNNaker simulations of the network state after 5 h. Matrix elements show the RCs (color bar) of all pairs the 800 excitatory model neurons in the simulation computed from 60 s of data. The order of the neuron ids in both symmetric matrices is determined by hierarchical clustering (Ward's variance minimization algorithm, details see Section 3.1.3) of the C matrix. Auto-correlations are set to 0 to not stretch the color scale.

Figure 8 displays the rate-correlation matrices of all excitatory neurons for the C and SpiNNaker simulation. The clustering arranges large correlation values close to the diagonal in the C result. A similar arrangement is not visible for the SpiNNaker result. Vice versa, a similar behavior is observed if the SpiNNaker data are used to cluster the neurons (not shown).

The similarity of the correlation structure is further quantified using the normalized scalar products of the RCs for the C and SpiNNaker simulation in the 5 network states, as described in Section 3.1.3. The resulting values range from 0.176 to 0.209. We assess the significance of the similarity by comparing the SpiNNaker data to 10,000 surrogate matrices computed by random permutations of the neuron identities. The mean of the surrogate scalar products reflecting structurally independent correlations range from 0.081 to 0.108. The observed score of the two implementations is thus at least 43 standard deviations away from the corresponding surrogate mean and indicates a similarity of correlation structures clearly beyond chance.

**Figure 9:**
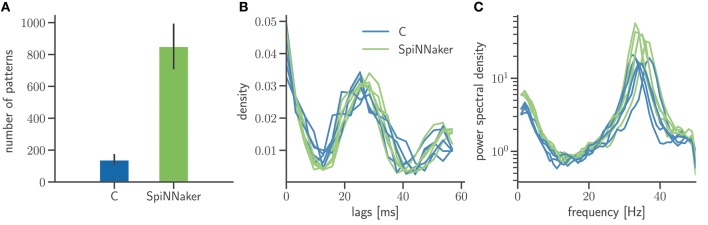
Frequency and structure of spatiotemporal spike patterns. **(A)** Bar diagram of the number of patterns detected using the SPADE method (Quaglio et al., 2017) in the two simulations. Displayed are the mean and standard deviation of the results for the 5 network states. The spike times of all 800 excitatory neurons are discretized by 3 ms bins and only spike sequences that repeated 3 or more times, are formed by at least 5 spikes, and with a temporal length between first and last spike shorter than 60 ms are considered. **(B)** Normalized distributions of the temporal lags between any two spikes involved in one of the patterns. The results for each of the 5 network states are displayed as a separate distribution. **(C)** Power spectra of the population activity in each network state. The spectra are calculated by Welch's method with a 100 Hz sampling frequency and a 1 Hz frequency resolution (window overlap: 50%). In all panels, data from the C and SpiNNaker simulations are indicated in blue and green, respectively.

In addition to mono- and bivariate statistics we analyze the spiking activity for both C and SpiNNaker simulation with SPADE (Quaglio et al., 2017) to detect spatiotemporal patterns (STPs) as potential dynamic signatures of the underlying network connectivity. In order to have the largest possible sample of patterns we consider all repeated spike sequences irrespective of their significance. This is justified as we are not interested in the significance of the results of the C and SpiNNaker simulation but in the comparison of the respective pattern formation. Figure 9 summarizes two characterizations of pattern occurrence: the total number of patterns and the temporal lags between the spikes forming a specific STP. While SpiNNaker shows a larger total number of patterns, the lag distributions are qualitatively similar in both simulations. Furthermore the power spectrum of the spiking activity pooled across all neurons (Figure 9C) exposes a clear peak around 35 Hz for both SpiNNaker and C, which explains the large number of lags around 27 ms in the patterns' lags distribution (Figure 9B). The phenomenon is enhanced in the SpiNNaker simulations, which exhibit both a larger average firing rate and a larger power around 35 Hz, explaining the larger number of spatiotemporal patterns.

## 5. Discussion

The study describes a workflow for the systematic, formalized and reproducible validation of network models based on the statistical comparison of the emerging neuronal activity. We show that a statistical approach is required, as not only the explicit model parameters but also the properties of the simulation engine affect the simulation outcome, leading in general to simulations that are not identical in their spike times. A quantitative comparison of model vs. experiment and of model vs. model is beneficial not only as a final validation but also guides the development process. The tests applied in our workflow span from monovariate (e.g., firing rates) to bivariate (e.g., correlation coefficients) to higher-order (e.g., spike patterns) statistical measures. Each measure of the spiking statistics reflects only a certain aspect of network activity. Therefore, the validation is enriched by including multiple measures to capture a broad range of network dynamics. The presented workflow is available online in an executable format with the intent to serve as a template and building block for validation tasks in computational neuroscience.

In conjunction with work presented in (Trensch et al., 2018) we assess as an example the implementation of the polychronization model by Izhikevich (2006) in the programming language C and on the neuromorphic hardware SpiNNaker. As the aim of this comparison is to validate the implementation of this model on SpiNNaker, we perform a model substantiation technique, where the C simulation assumes the role of the reference model.

Initially, the quantitative comparison of characteristic measures of the population dynamics (Section 4.1) exposes an artifact. The artifact originates from an overflow of the SpiNNaker fixed-point data type that is caused by an inappropriate detection of threshold crossing (see Trensch et al., 2018, for details) leading to several overactive neurons that sporadically enter phases in which they fire in every simulation time step. Thus, rigorous validation testing in the iterative model development process is useful already in early stages because it uncovers mismatches also in simple measures and complements the model verification.

Further refinement of the ODE solvers for both model implementations leads to an improved agreement of FR and CC, but increases the discrepancy of the distributions of LVs between the C and SpiNNaker implementation (Figure 6). This intermediate result emphasizes the importance of considering multiple statistics in parallel throughout the validation process as each statistic highlights different dynamic characteristics of the underlying model. The example also demonstrates that statistics of higher order (here pairwise correlations) are not necessary informative of differences in the network activity captured by lower order statistics (here monovariate LV). Therefore, a sufficient agreement in the statistics of a given order does not imply sufficient agreement in the statistics of lower order.

Subsequent analysis traces the discrepancy in the LV measure back to a software issue causing a small delay in spike timing. Solving this issue in the final iteration step leads to a satisfactory agreement between the C and SpiNNaker implementations in terms of the effect sizes of the different statistical measures (Section 4.3). An analysis of the spatiotemporal structure of the spiking activity in the network shows that the temporal structure (lag distributions) of spike patterns found in the data is qualitatively similar for the two implementations. However, the dominant elements of the correlation structure (in the sense of strong intra-correlated groups of neurons) cannot be attributed to the same neurons in the two simulations (Figure 8). Statistical hypothesis tests for equality of the mean (*t*-test) and equality of the distributions (Kolmogorov-Smirnov, Mann-Whitney *U* test) failed for all statistics except the distribution of eigenvalues. Taken together, the complexity of these findings emphasizes the importance of using multiple statistical tests to obtain a complete understanding of the validation outcome.

Quantifying the similarity between the simulations is not the final step of validation. It has to be evaluated whether or not this similarity (or, range of accuracy) represents an acceptable agreement with respect to the intended application of the respective models. This evaluation requires consideration of the requirements and intentions of the application. Conversely, the statistical agreement obtained in the validation process defines the applicability and accuracy of the model. Following the latter approach, this study quantifies the accuracy of the SpiNNaker implementation. Strong requirements for the SpiNNaker simulations, such as an equal number of patterns found with SPADE or the statistical equivalence of the calculated distributions as assumed by the null hypothesis of typical two-sample tests, can so far not be fulfilled. This means that the acceptable agreement is not yet reached for analyses with strong statistical requirements. With the intention of achieving a qualitative reproduction of Izhikevich's polychronization model, however, we can state that the final model implementation on SpiNNaker is in acceptable agreement with the corresponding C simulation. An alternative end of the iterative validation loop occurs when remaining discrepancies are understood and result from the intrinsic limitations of the underlying simulation technology (e.g., the SpiNNaker neuromorphic hardware and its software stack). Particularly, experimental electrophysiological recordings often contain considerable variability (see e.g., Arieli et al., 1996; Mochizuki et al., 2016; Riehle et al., 2018, for trial-to-trial and subject-to-subject variability). Therefore, the acceptable agreement for a model to explain relevant experimental data may in some cases be formulated less strictly, e.g., in terms of effect sizes that reflect the typical variability between multiple equivalent data sets.

The framework implemented by NetworkUnit can also be used for such a quantitative comparison of two experimental data sets. To illustrate this, we developed a second worked example showing the statistical quantification of the difference between two published experimental data sets (Brochier et al., 2018) obtained in the motor cortex of two macaque monkeys. The detailed and fully documented analysis can be found online^18^. In summary, we find that the spike statistics, evaluated on the basis of the FR, ISI, and LV measures, are significantly different between the two monkeys, but exhibit effect sizes below 1. However, care must be taken in interpreting such comparisons of experimental data due to the large number of factors contributing to the observed variability.

The question of the required accuracy in the representation of parameters of the model (e.g., synaptic weights) could be further investigated using the tools presented in the work. Thus, further development of the neuromorphic hardware while continuously reapplying the verification and validation tests outlined in this paper and in Trensch et al. (2018) may lead to a more accurate implementation that will widen the range of applications.

The statistical tests and tools for quantitative comparison are realized within the open source framework of the Python module NetworkUnit. It is based on SciUnit, a module designed for scientific model validation (Omar et al., 2014). The aim of NetworkUnit is to provide a battery of tests applicable to compare network activity from spiking neural network models. As such, its intent is to provide a formal structure and standard implementations for validation tests to simplify even complex validation scenarios, such as the successful port of the cortical microcircuit model (Potjans and Diesmann, 2014) to SpiNNaker described by van Albada et al. (2018). Indeed, the process of defining validation workflows and corresponding performance indices to evaluate accuracy and usefulness has common practice in other computational disciplines, such as climate research (Feichter, 2011), and represents a core component in large-scale modeling efforts, such as the Human Brain Project.

The presented workflow and the tests can be easily adapted to a range of other validation and substantiation scenarios, including the comparison to experimental data, to other models, but also the quantitative comparison of different experimental data sets, e.g., to test for inter-subject consistency. Network-level validation is in principle not even restricted to a specific format of activity. Since we here evaluate a spiking network model all tests of this study are based on the model capability to produce spike trains. However, the evaluation of models which predict continuous activity signals such as LFP, MEG, or EEG, is equally tractable using tests that are based on the corresponding capability (i.e., to produce corresponding signals). NetworkUnit can be further extended to include different statistical measures and statistical hypothesis tests in order to account for user-specific validation scenarios of simulated and/or experimental results. Other examples include the separate analysis of subpopulations such as inhibitory and excitatory units and the question of how the biophysical complexity of neuron models influences the emerging network dynamics. A note of care, however, has to be issued concerning the interpretation of tests performed on subpopulations of a network, where its quantified evaluation will most likely be contingent on the detailed dynamics exhibited by the other populations.

The continued evolution of such concepts and software components to rigorously define and formalize the validation process is a key step to increase the confidence in models developed by the neuroscience community, and ultimately leads not only to more replicability, but also true reproducibility of scientific findings.

## Software and Data Resources

The data and the code to perform the analysis presented in this study can be found at https://web.gin.g-node.org/INM-6/network_validation (doi: 10.12751/g-node.85d46c). Validation testing was performed using the NetworkUnit Python module available at https://github.com/INM-6/NetworkUnit. All data analysis, including the SPADE method, was performed using the Elephant Python package http://python-elephant.org.

## Author Contributions

RG, MvP, GT, SG, and MD designed the study. RG and PQ performed the analysis. GT performed the simulations and implemented the model. RG, MvP, and PQ wrote the software for performing the validations. RG, MvP, PQ, GT, SG, and MD contributed to writing of manuscript.

### Conflict of Interest Statement

The authors declare that the research was conducted in the absence of any commercial or financial relationships that could be construed as a potential conflict of interest.

## Footnotes

^1^https://www.alleninstitute.org

^2^https://www.humanbrainproject.eu

^3^https://github.com/AllenInstitute/bmtk

^4^https://github.com/Neurosim-lab/netpyne

^5^https://github.com

^6^http://opensourcebrain.org

^7^https://bbp.epfl.ch/nmc-portal

^8^https://gin.g-node.org

^9^https://senselab.med.yale.edu/modeldb

^10^https://neuroelectro.org

^11^https://crcns.org

^12^https://github.com/simetenn/uncertainpy

^13^https://github.com/INM-6/NetworkUnit

^14^https://web.gin.g-node.org/INM-6/network_validation

^15^http://python-elephant.org

^16^https://www.izhikevich.org/publications/spnet.htm

^17^https://github.com/gtrensch/RigorousNeuralNetworkSimulations (doi: 10.5281/zenodo.1435831)

^18^https://web.gin.g-node.org/INM-6/network_validation/src/master/NetworkUnit_examples.ipynb
